# Profiles of Attendees in Voluntary Counseling and Testing Centers of a Medical College Hospital in Coastal Karnataka

**DOI:** 10.4103/0970-0218.39243

**Published:** 2008-01

**Authors:** S Jayarama, Shaliny Shenoy, B Unnikrishnan, John Ramapuram, Manjula Rao

**Affiliations:** Department of Community Medicine, Kasturba Medical College Hospital, Mangalore, Karnataka, India

**Keywords:** HIV, voluntary counseling and testing center, high-risk sexual behavior

## Abstract

**Research Question:**

What are the socio-demographic profile and risk behavior pattern of seropositive attendees in the voluntary counseling and testing center (VCTC)?

**Study Design:**

Retrospective study.

**Setting:**

VCTC in the outpatient complex of Kasturba Medical College Hospital, Mangalore, Karnataka.

**Subjects:**

Records pertaining to all the 539 and 330 seropositive attendees during the years 2005 and 2006, respectively, were included in the study besides data from 2001 onwards in order to assess the time trend of human immunodeficiency virus (HIV).

**Study Variables:**

Age, sex, marital status, religion, educational status, occupation, place of residence and pattern of risk behavior in relation to HIV/AIDS.

**Statistical Analysis:**

Analysis was done with SPSS version 11. Statistical test and Chi-square was done, and *P* < 0.05 was considered statistically significant.

**Results:**

The time trend of VCTC attendees reveals a gradual increase except in 2006 showing a sharp decline. Seropositives were around 20% between 2001 and April 2007 with a sharp increase in 2006, i.e., 33.64%. Male seropositivity constituted 60-63%; 81-91% of seropositive attendees belonged to the age group of 15-50 years; 58-70% were married. Only about 3% were illiterates and 20-25% constituted 6^th^-12^th^ pass-outs. With regard to occupational profile, about 17-27% were housewives, 19-21% were laborers/hotel workers and 7% were entrepreneurs. About 45% were from urban area and nearly one-third hailing from other districts in the border of Karnataka. About 25% were exposed to commercial sex workers; another 21-23% were involved in premarital sex and nearly 38% were indulging in heterosexual activities.

## Introduction

From the detection of this mysterious illness for the first time in 1986 at Chennai, the HIV infection has been growing very fast in India. The number of people living with HIV/AIDS was estimated to be 5.1 million by December 2001.(1) The distribution and spread of the disease is rather uneven in India. The assessment of socio-demographic factors, level of awareness as well as risk behavior of the population is mandatory in order to plan interventional strategies. The voluntary counseling and testing center (VCTC) is an entry point to care and support services, which provides people with an opportunity to know and understand HIV serostatus in confidential manner.(2) The data generated by VCTC may provide important clues to understand the epidemiology of the disease in a particular part of the country. Voluntary counseling and testing could be considered one of the cost-effective way of reducing HIV transmission in resource-poor countries.(3)

## Materials and Methods

The present study was conducted among the attendees of VCTC of Kasturba Medical College (KMC) Hospital attached to the outpatient complex instead of the Microbiology Department of the college for its optimal utilization. Since comprehensive HIV/AIDS control program was being done much before the various governmental programs, this VCTC was one among the first five to be established in Karnataka during April 2001. Their experience can be a model for the benefit of newer VCTCs/integrated counseling and testing centers (ICTCs).

The VCTC in KMC is a part of infectious diseases cell where complete and comprehensive care (diagnosis, counseling and management of opportunistic infections) is provided to HIV/AIDS patients; it can aptly be called Provider Initiated Testing and Counseling Center. The VCTC also has a vibrant positive people network that meets at least once a month.

The study included all those who were referred/walking in to the center during the years 2005 and 2006. Anonymous data from the relevant records of VCTC were collected by a pretested structured proforma in conformity with National AIDS Control Organization (NACO) guidelines(4) Blood samples were collected and tested by three rapid/spot tests by lab technicians under strict supervision(4–6) of a designated microbiologist from the Department of Microbiology. Those persons whose tests showed negative on aids tests were given post-test counseling and declared seronegative. Those samples showing positive were subjected to Papillus test and later Tridot test. Persons showing negative in the second and/or third tests were advised to come after 4-8 weeks for review. Data were analyzed using SPSS version 11. Chi-square was done and *P* < 0.05 was considered statistically significant.

## Results and Discussions

### Trend of VCTC attendees and their seropositivity status

The trend of VCTC attendees [Table 1] reveals a gradual increase except in 2006, wherein there was a sharp decline possibly after setting up VCTCs and ICTCs at peripheral health institutions including the one at the nearby district government hospital. In addition, seropositivity was around 20% in all these years with a sharp increase in 2006, i.e., 33.64% (368 out of 981). A possible explanation to this trend was the confidence in the clientele of the VCTC in particular and KMC/Manipal in general. Another prominent finding was the optimal utilization by females (nearly 40%) of this center, which is located in the outpatient premises of the hospital, in comparison with 26% users in a Darjeeling study.(7) In a study(8) conducted in a rural community of northern Thailand, HIV prevalence was found to be 4.9%. In another study(9) conducted in a VCTC of a district hospital of Thailand, HIV prevalence was found to be 29%; it showed an increasing trend from 1995 to 1997 when the prevalence was 39%, which declined to 28% in 1999. To increase the number of HIV testing, the VCTC conducts outreach camps (once a week) in industries with truck drivers, auto drivers and taxi drivers. In colleges, awareness programs are conducted and voluntary testing is also done after obtaining written informed consent.

**Table 1:** Trend of seropositivity amongst attendees of VCTC clinic seropositives

Year	Male no. (%)	Female no. (%)	Total no.	Male	Female	Total no. (%)
2001	-	-	608	94	63	157 (25)
2002	1086 (56.95)	827 (43.05)	1907	221	138	359 (19)
2003	1052 (59.91)	704 (40.09)	1756	246	149	395 (22)
2004	1367 (59.20)	942 (40.80)	2309	293	183	476 (21)
2005	1611 (67.21)	786 (32.79)	2397	364	175	539 (23)
2006	591 (60.24)	390 (39.76)	981	209	121	330 (34)
2007 (till April)	1100 (60.34)	723 (39.66)	1823	246	151	397 (22)

### Age and sex profiles

The distribution of seropositive attendees by their age and sex [Table 2] reveals that males constituted 67.53% in 2005 and 63.33% in 2006. In both the years, 15-50-year age group constituted 90.86% and 80.99%, respectively, which is in conformity with the national figure.(4,10) Among 0-5-year group, seropositivity was 4.57% in 2005 and 2.48% in 2006. However, both age and sex distribution patterns were not statistically significant. A Thailand study(9) found that the socio-demographic characteristics like men between age 20 and 49 years and women less than 16 years were significantly associated with HIV prevalence. In a study conducted in Croatia, a majority of the seropositive clients were between 25 and 29 years old.(11)

**Table 2:** Age and sex profile of seropositive attendees

Age Year	2005 (*n* = 539)		2006 (*n* = 330)	
		
	Male no. (%)	Female no. (%)	Male no. (%)	Female no. (%)
0-5	10 (1.20)	8 (4.57)	3 (1.44)	3 (2.48)
6-14	04 (1.20)	4 (2.28)	5 (2.39)	6 (5.96)
15-50	335 (92.03)	159 (90.86)	178 (85.17)	98 (80.99)
50+	13 (3.56)	4 (2.28)	18 (08.61)	3 (2.48)
Not specified	2 (0.55)		5 (2.39)	11 (9.09)
Total	364 (67.53)	175 (32.47)	209 (63.33)	121 (36.66)
	χ^2^ = 2.927, *P* = 0.403		χ^2^ = 0.429, *P* = 0.0925	

### Profile of marital status

The distribution of seropositive attendees as per marital status [Table 3] reveals that “married” people constituted 57.70% and 70.30%, respectively, during the years 2005 and 2006, which is similar to a Darjeeling study.(7) The “separated” were 4.08% and 11.52%, respectively, during the same period. This change in composition with higher percentage of attendees married as well as separated was statistically very highly significant. In a Thailand study(9) as well, divorcees and widows were found more likely to be HIV positive

**Table 3:** Demographic profiles of HIV positives attending VCTC

Characteristics	2005 (*n* = 539) No. (%)	2006 (*n* = 330) No. (%)	Chi-square	*P*-value
Marital status			89.9	*P* < 0.0001
Married	311 (57.7)	232 (70.3)		
Unmarried	206 (38.2)	60 (18.2)		
Separated/divorce	22 (4.1)	38 (11.5)		
Religion			21.1	*P* < 0.0001
Hindu	444 (82.4)	232 (70.4)		
Muslim	56 (10.4)	53 (16.1)		
Christian	38 (7.1)	39 (11.8)		
Others	1 (0.2)	6 (1.8)		
Education			15.8	*P* < 0.001
Illiterate	14 (2.6)	10 (3)		
Primary	313 (58.1)	197 (59.7)		
6-12 standard	137 (25.4)	66 (20)		
Graduate	5 (0.9)	16 (4.9)		
Others	70 (13)	41 (12)		
Residence			14.6	*P* < 0.05
Mangalore tehsil	258 (45.9)	132 (40)		
Bantwal (rural)	39 (7.2)	24 (7.2)		
Belthangady (rural)	34 (6.3)	20 (6.1)		
Puttur (rural)	20 (3.7)	11 (3.3)		
Sullia (rural)	8 (1.5)	6 (1.8)		
Udupi district	52 (9.7)	21 (6.4)		
Kasargod district	70 (1.3)	70 (21.2)		
(Kerala) Other districts	58 (10.8)	40 (12.1)		
Occupation			36.8	*P* < 0.0001
House wife	93 (17.3)	89 (27)		
Laborer/hotel worker	111 (21)	63 (19.1)		
Driver	22 (4.1)	10 (3)		
Students	7 (1.3)	9 (2.7)		
Beedi roller	32 (5.9)	20 (6.1)		
Agriculture	15 (2.8)	0 (0)		
Business	37 (6.9)	25 (7.6)		
Semi-skilled	24 (2.5)	30 (9.1)		
Others	198 (36.7)	84 (25.5)		

### Religion profile

It was observed [Table 3] during the years 2005 and 2006 that Hindus constituted 82.37% and 70.38%, respectively. This pattern was proportionate to the distribution among all religions and was statistically very highly significant. Thus, in spite of religion/moral conducts, seropositivity is a problem among all religions requiring concerted interventional efforts.

### Education profile

Education profile of seropositive attendees [Table 3] reveals that primary education group constituted 58% and 60% during the years 2005 and 2006, respectively. The illiterate group constituted only 2.6% and 3% during the same period. However, despite the higher literacy state, coastal population is showing higher seropositivity and the same is statistically significant. This may be due to their inadequate awareness about reproductive health and STDs. At present, sex education is not included in our secondary school curriculum and there may be a need for the same.

### Place distribution of seropositive attendees

Place distribution of seropositive attendees [Table 3] reveals that urban area (Mangalore) constituted 45.87% and 40% during the years 2005 and 2006, respectively. From outside the district and the neighboring state of Kerala, it constituted 33.4% and 39.70% during the same period. The low seropositivity among semi-urban and rural population of the district may be due to opening of newer VCTCs during the year 2005 at community health centers and tehsil hospitals. Higher positivity among the attendees outside the district may be due to the confidence of the population with the VCTC functioning in the KMC/Manipal group hospital premises as various HIV/AIDS prevention and control programs are being earnestly implemented since many years.

### Occupational profile of seropositive attendees

Occupational profile of seropositive attendees [Table 3] reveals varied distribution among laborers and hotel workers (20.59% in 2005 and 19.9% in 2006). Similarly, housewives constituted 17.25% and 26.97% during the same period; beedi rollers, business and semi-skilled professionals constituted a sizeable number. Drivers constituted only 4.08% and 3.08%, respectively, in comparison to 13% with Darjeeling study.(7) This less reporting by drivers may be possibly due to the location of VCTC in the heart of the city. Highway truckers are mainly utilizing the static as well as mobile VCTCs run by an NGO Population Services India (PSI), Mangalore, with financial assistance from foreign NGOs and NACO India.

### Pattern of risk behavior

The pattern of risk behavior of seropositives especially among males [Table 4] reveals nearly 72-77% contracted HIV through sexual contact, nearly 25%, almost all males, had exposure to commercial sex workers (CSWs) and nearly 38%, almost all males, had multiple sex partners; premarital sexual exposure was up to 21-23 %, mostly among males. It was found that (46 out of 364) 12% of the spouses of male attendees were positive in comparison to 34% (59 out of 175) spouses of female attendees, which was found to be statistically significant; similar pattern was also observed for the year 2006. This corroborates with Darjeeling study(8) wherein more than two-thirds of male attendees were clients of CSWs and more than half of seropositive males were enjoying married life, thus spreading to their spouses. About 5-6% were from parent-to-child transmission and this number may increase progressively. Only 0.37-1.21% were from blood transmission, which may be due to a successful blood safety program of Government of India. In another study done in India, among HIV positive defense service corps personnel around 95.6% acquired the disease through heterosexual mode from CSWs.(12)

**Table 4:** Pattern of risk behavior of seropositives attending voluntary counseling and testing center

	2005 (*n* = 539)			2006 (*n* = 330)		
		
Route of transmission	Male *n* = 364	Female *n* = 175	Total no. (%)	Male *n* = 209	Female *n* = 121	Total no. (%)
Sexual	232	156	388 (72)	135	120	255 (77)
Exposed to CSWs	130	5	135 (25)	83	1	84 (25)
Exposure to multiple sexual partners	202	6	208 (39)	122	4	126 (38)
Premarital sex	109	4	113 (21)	75	1	76 (23)
Spouse HIV +ve*	46	59	105 (19)	40	50	90 (27)
Parent to child	14	13	27 (5)	11	9	20 (6)
Received blood	1	1	2 (0.3)	4	0	4 (1.2)
Injecting drugs/unknown	19	10	29 (5.4)	26	6	32 (10)

Many attendees had more than one high-risk sexual behavior

* *P* < 0.001

Based on the VCTC reports and findings, targeted interventions are carried out among CSWs like creating awareness, condom distribution, etc. by NGOs, namely Hind Kushta Nivaran Sangh in coordination with the VCTC.

## Conclusion

The findings of the present study carried out at the medical college hospital reveal that (except for the inadequate data from semi-urban/rural population) in spite of social stigma attached to HIV, the reporting and care-seeking behavior of the local population including females of HIV suspects is very high. The pattern/risk behavior especially multiple sex with CSWs with substantial percentage indulging in premarital sex and multiple sexual partners is a cause for concern requiring appropriate remedial measures. Although it appears to be an Herculean task in view of the rising trend and unabated spread of HIV to the general population through the bridge population, to achieve zero level of growth of HIV/AIDS by 2007,(1) no time should be wasted to carry out priority targeted interventions among the selected high-risk groups of coastal area by financing all those private institutions (in addition to recognized NGOs) carrying out earnestly HIV/AIDS control/care services.

Intense IEC activities including involvement of electronic media, involvement of religious heads, etc. may have to be done to promote behavioral changes for the better. Policy authorities, particularly NACO, besides depending on the sentinel surveillance can also take relevant data from certain well-run VCTCs in order to implement various socio-health measures to control not only spread of HIV and AIDS but also in supporting the people living with HIV/AIDS (PLWHAS).
